# Full Length Interleukin 33 Aggravates Radiation-Induced Skin Reaction

**DOI:** 10.3389/fimmu.2017.00722

**Published:** 2017-06-28

**Authors:** Olga Kurow, Benjamin Frey, Louis Schuster, Verena Schmitt, Susanne Adam, Madelaine Hahn, Derek Gilchrist, Iain B. McInnes, Stefan Wirtz, Udo S. Gaipl, Gerhard Krönke, Georg Schett, Silke Frey, Axel J. Hueber

**Affiliations:** ^1^Department of Internal Medicine 3—Rheumatology and Immunology, Friedrich-Alexander-Universität Erlangen-Nürnberg (FAU), Universitätsklinikum Erlangen, Erlangen, Germany; ^2^Department of Radiation Oncology, Universitätsklinikum Erlangen, Friedrich-Alexander-Universität Erlangen-Nürnberg (FAU), Erlangen, Germany; ^3^Division of Infection, Immunity and Inflammation, Glasgow Biomedical Research Centre, University of Glasgow, Glasgow, United Kingdom; ^4^Department of Medicine 1, Universitätsklinikum Erlangen, Friedrich-Alexander-Universität Erlangen-Nürnberg (FAU), Erlangen, Germany

**Keywords:** skin inflammation, interleukin-33, dermatitis, radiation, necrosis

## Abstract

The interleukin (IL)-1 family member IL-33 has been described as intracellular alarmin with broad roles in wound healing, skin inflammation but also autoimmunity. Its dichotomy between full length (fl) IL-33 and the mature (m) form of IL-33 and its release by necrosis is still not fully understood. Here, we compare functional consequences of both forms in the skin *in vivo*, and therefore generated two lines of transgenic mice which selectively overexpress mmIL-33 and flmIL-33 in basal keratinocytes. Transgene mRNA was expressed at high level in skin of both lines but not in organs due to the specific K14 promoter. We could demonstrate that transgenic overexpression of mmIL-33 in murine keratinocytes leads to a spontaneous skin inflammation as opposed to flmIL-33. K14-mmIL-33 mice synthesize and secrete high amounts of mmIL-33 along with massive cutaneous manifestations, like increased epidermis and dermis thickness, infiltration of mast cells in the epidermis and dermis layers and marked hyperkeratosis. Using skin inflammation models such as IL-23 administration, imiquimod treatment, or mechanical irritation did not lead to exacerbated inflammation in the K14-flmIL-33 strain. As radiation induces a strong dermatitis due to apoptosis and necrosis, we determined the effect of fractionated radiation (12 Gy, 4 times). In comparison to wild-type mice, an increase in ear thickness in flmIL-33 transgenic mice was observed 25 days after irradiation. Macroscopic examination showed more severe skin symptoms in irradiated ears compared to controls. In summary, secreted mmIL-33 itself has a potent capacity in skin inflammation whereas fl IL-33 is limited due to its intracellular retention. During tissue damage, fl IL-33 exacerbated radiation-induced skin reaction.

## Introduction

The cytokine interleukin 33 (IL-33) is a member of the IL-1 family contributing to pathogenesis in allergic lung diseases (1, 2), atopic dermatitis (3), sepsis (4), inflammatory tendinopathy (5) but also to rheumatoid arthritis (6) and psoriasis (7). IL-33 is constitutively expressed in epithelial cells from tissues with a barrier function, as well as in endothelial cells and fibroblasts (8). Upon various types of endothelial or epithelial cell damage, IL-33 is released and binds to the heterodimeric receptor complex ST2L/IL1-RacP expressed on Th2 and diverse types of innate immune cells (9–11). IL-33 functions *via* two ways: firstly, IL-33 is a nuclear cytokine with restricted nuclear localization (12). In the nucleus, due to a helix turn helix like motif, IL-33 condenses chromatin (13) and has been suggested to suppress pro-inflammatory gene transcription (14). Secondly, as alarmin, released during cell damage, IL-33 activates the immune system (12, 15). Apoptosis deactivates IL-33 whereas necrosis provides IL-33 as a bioactive protein. During apoptosis, IL-33 will be cleaved by activated caspase 3 and 7 in the IL-1-like cytokine domain. This short form of IL-33 has an attenuated biological activity and no capacity to activate the immune system (16). Upon necrotic cell death or mechanical injury, the full length form of IL-33 (fl IL-33) is released in the extracellular space (15, 16). The inflammatory proteases (neutrophil serine proteases, cathepsin G, and elastase) play an important role in the maturation process of fl IL-33 leading to a shorter mature form with increased biological activity (17). Interestingly, active externalization of IL-33 has been described by stimulation with proinflammatory cytokines such as tumor necrosis factor-alpha (TNF-α) (18).

So far, effector function of IL-33 is still under discussion, and multiple models of diseases have been described to be influenced by IL-33 signaling. In this notion, stimulation with IL-33 has been demonstrated to activate T-helper (Th)1, Th17, or Th2 immune response (19). In models of lung inflammation, IL-33 induced Th2 cytokines such as IL-5 and IL-13, as well as elevated levels of IL-1, IL-4, IL-6, IL-10 (20, 21). A mouse model with local overexpression of IL-33 in keratinocytes led to Th2 induced dermatitis (3). In contrary, under defined conditions, IL-33 can induce a Th1 immune response thereby producing Th1-type cytokines by natural killer and NKT cells (19, 22). Moreover, in Th1- and Th17-driven models of arthritis or psoriasis, IL-33 plays a proinflammatory role (7, 13, 21, 23).

In humans, IL-33 has been implicated in allergic inflammation such as asthma (24, 25) and atopic dermatitis (3, 26). In psoriatic skin increased IL-33 expression was detected on transcriptional (mRNA) and protein level but not in atopic dermatitis lesions or normal human skin (7, 18). Additionally, anti-TNF-α therapy downregulated IL-33 mRNA expression in skin of psoriatic patients (18).

Here, we wanted to elucidate the role of IL-33 in skin inflammation using different IL-33 expression models. Our work revealed a high skin inflammatory potential of IL-33 with an “alarmin” function during radiation.

## Materials and Methods

### Mice

C57Bl/6 wild-type (WT) mice were obtained from Charles River Laboratories Sulzfeld, Germany. hK14mIL33tg were generated on a C57Bl/6 background (see below). KRT14-cre (CD1 background) mice were obtained from Jackson Laboratory (Bar Harbor, ME, USA). The mmIL-33-GFP-CRE mice (C57Bl/6 background) were kindly provided by Dr. S. Wirtz (27).

### Construction of the K14-IL-33 Transgene

The transgene is expressed in a K14 expression vector (28). It consists of a 2-kb human keratin 14 promoter (Gen-Bank accession no. DQ343282.1), a 0.66-kb rabbit-beta globin intron followed by a 0.8-kb full-coding sequence of mouse IL-33 cDNA (Gen-Bank accession no. NM_133775.2) and a 0.35 kb human keratin 14 poly(A) signal DNA fragment. For generation of the vector encoding a secreted version of IL-33 (mmIL-33) controlled by skin-specific regulatory elements, we inserted mouse mature IL-33 cDNA into the K14 vector (27).

### Transient Transfection

The human keratinocyte cell line HaCaT (#300493, CLS Cell Lines Service, Germany) and the murine keratinocyte cell line PDV were cultured in Dulbecco’s modified Eagle’s medium supplemented with 10% heat-inactivated fetal bovine serum (Invitrogen, Carlsbad, CA, USA), 100 U/ml penicillin and 100 mg/ml streptomycin. Cells were maintained at 37°C and 5% CO_2_. The K14 expression vector was transiently transfected into HaCaT cells using Lipofectamine 2000 transfection reagent (Invitrogen, Carlsbad, CA, USA) according to the manufacturer’s instructions. Murine keratinocytes were transfected using FuGENE^®^ HD Transfection Reagent according to the manufacturer’s instruction (Promega, Madison, WI, USA).

### Western Blot

After collecting the supernatant, all cells were pelleted, washed with cold phosphate-buffered saline (PBS), and lysed in RIPA buffer. Before denaturation with Laemmli buffer, protein concentration adjustment was conducted. Supernatants were resuspended in 6× Laemmli and boiled at 98°C for 10 min. Cell pellets and supernatants were separated on a 10% SDS-polyacrylamide gel. Membranes were blocked with 5% non-fat dry milk (Roth, Karlsruhe, Germany), probed with mouse anti-human IL-33 antibody (1:1,000; Nessy-1, Enzo Life Sciences, Lörrach, Germany), and peroxidase-labeled anti-mouse as secondary antibody (Dako, Denmark). Membranes were developed using the enhanced chemiluminescence method.

### Purification of Plasmid for Pronuclear Injection

Linear transgene K14-IL-33 DNA fragment was separated from vector backbone by gel electrophoresis. The K14-IL-33 fragment was cut out, transferred in a dialysis bag (Spectra/Por MWCO 3500, Spectrumlabs, DG Breda, The Netherlands), filled with running buffer (1× TAE), and closed with clamps. After electrophoresis for 1 h at 80 V, 90% of the DNA was eluted. The DNA was purified by Elutip-D minicolumns from Schleicher & Schuell (Dassel, Germany).

### Generation of Transgenic Mouse Lines

The generation of transgenic mice by pronuclear injection of K14-IL-33 transgene was performed by the Transgenic Mouse Facility, Friedrich-Alexander-Universität Erlangen-Nürnberg, Germany. Of 16 pups born, 2 mice were positive for the transgene. The transgenic founders were identified by PCR for expression of the K14 promoter (IL-33-seq7fw 5′ CAGTTGATCCCAGGAAGAGC 3′ and IL-33-seq6rev GCAGGCTACACTTTCCCATC) and K14-IL-33 transgene (IL-33-seq7fw CAGTTGATCCCAGGAAGAGC and IL-33-seq2rev GTTGCAGCTCTCATCTTTCTCC) and by quantitative real-time PCR for the expression of mouse IL-33 using the primer pair: K14IL-33int/ex fw CTGCAAGTCAATCAGGCGAC and K14IL-33 int/ex rv TGCAGCCAGATGTCTGTGTC. A mouse line that highly overexpressed IL-33 was generated from these mice by breeding with littermates. To establish K14-CreER™/GFPmmIL33 mice, homozygous K14-CRE™ mice were crossed with heterozygous tamoxifen inducible mmIL-33-GFP-CRE mouse. The offspring were tested for mmIL-33 expression using flow cytometric analysis of blood for GFP expression (Figure 3B) thus using negative littermate animals as control mice for all experiments.

All protocols used in these studies were in compliance with federal guidelines and the Amgen Institutional Animal Care and Use Committee. All mice were maintained in a SPF facility. All animal experiments were approved by the animal welfare committee and approved by the “Regierung Unter-/Mittelfranken.”

### *In Vivo* Imiquimod (IMQ) Treatment

The right ears of both WT and hK14mIL33tg mice at 8–10 weeks of age were topically treated with an IMQ-containing cream (Aldara, MEDA Pharma) for 7 consecutive days. Control ears were treated similarly with a vehicle cream. Ear swelling was measured daily before treatment. On day 7, after sacrificing the mice, ears were collected for H&E, immunohistochemistry (IHC) and analyzed for epidermal thickness.

### Acute Barrier Disruption Procedures

Barrier disruption due to removement of corneocytes was induced by skin stripping as reported previously (29). On day 3 after treatment, back skin of WT and hK14mIL33tg (Tg) were collected for immunohistochemical staining for IL-33, H&E and analyzed for epidermal thickness (mm).

### IL-23-Dependent Skin Inflammation Model

500 ng IL-23 (R&D Systems, Minneapolis, MN, USA) in a total volume of 20 µL was intradermally injected in the ears from WT and hK14mIL33tg (Tg) every other day. Sterile PBS was used as a vehicle control. Ear thickness was monitored before each injection. On day 15, ears were analyzed for epidermal thickness and collected for immunostaining.

### *In Vivo* Radiation

Dermatitis was induced by locally and fractionated irradiation of the right ear similar to a procedure used for mouse tumor irradiation with slight modifications (30). For irradiation, mice were anesthetized by isoflurane inhalation and placed in a special manufactured plexiglas^®^ box. During the irradiation procedure, the mice were kept under isoflurane inhalation anesthesia to avoid movement of the mice. The mice were locally irradiated with four single fractions of 12 Gy (cumulative dose of 48 Gy) only at the right ear. The irradiation field of the 6 MV linear accelerator (PRIMART, Siemens, Munich, Germany) was minimized, and irradiation was performed tangentially to preserve the head of the mice. This procedure was performed at days 1, 3, 5, and 7. Starting on day 2 and every second day thereafter, animals were scored in a blinded manner for ear thickness and dermatitis. Dermatitis scoring of all animals was performed analog to the Cancer Therapy Evaluation Program scale (31, 32). Briefly, dermatitis scoring ranged from: 0 = normal, no changes; 1 = mild erythema; 2 = moderate to severe erythema, slight desquamation; 3 = desquamation of 25–50% of irradiated area; 4 = desquamation of >50% of irradiated area; to 5 = frank ulcer.

### *In Vitro* Irradiation

K14 fl IL-33 transfected murine keratinocytes (PDV) were incubated at 37°C, 5% CO_2_ in humidified air in 24 well, flat-bottom plates. Ionizing irradiation was performed with an X-ray generator (120 kV; GE Inspection Technologies, Hürth, Germany). The dose rate was 8 Gy/min. Irradiated and control keratinocytes monolayer cultures were immediately returned to the incubator at 37°C in a humidified environment and cultured for 6–48 h. Supernatant and cells were analyzed by ELISA and western blot to assess the presence of the IL-33 cytokine.

### Tamoxifen Preparation and Administration

Tamoxifen (Cayman Chemical, Ann Harbor, MI, USA) was dissolved in an ethanol/DMSO mixture (equal parts) solutions at 100 mg/ml. Tamoxifen solution was freshly prepared the day prior to each administration and placed on a rolling device to dissolve overnight at room temperature. Before treatment, excess fur was shaved from the backs of recipient mice, which were topically treated with 20 mg Tamoxifen (200 µl volume).

### Histology and IHC

Mouse skin and ear samples were fixed in 4% formaldehyde and stained with hematoxylin and eosin. Epithelial hyperplasia was assessed using a Zeiss Axio Lab.A1 light microscope (Zeiss, Oberkochen, Germany) and quantified by measuring the mean distance from the *stratum basale* to the bottom of the *stratum corneum* in a blinded manner. Mast cells were stained by toluidine blue, numbers counted per full skin section and standardizing for the tissue area of different sections [using image analysis system (OsteoMeasure; OsteoMetrics, Decatur, GA, USA)].

For IHC, 8-µm paraffin sections were incubated with anti-mouse IL-33 antibody (Enzo Life Sciences, Lörrach) overnight at 4°C. Tissues were subsequently labeled with biotinylated goat anti-mouse IgG antibody (Vector Laboratories, Burlingame, CA, USA) at room temperature for 1 h, following streptavidin-HRP (Dako, Denmark) and DAB Chromogen (Dako, Denmark) incubation. Positive staining developed as a brown reaction.

### Quantitative Real-time PCR Analysis

Total RNA was isolated using peqGold TriFast from Peqlab (Erlangen, Germany). RNA was reverse transcribed using the MultiScribe™MuLV reverse transcriptase (Invitrogen, Thermo Fisher Scientific Inc., Waltham, MA, USA). Relative gene expression was measured with Sybr Green RT mix by quantitative real-time PCR using β-actin as endogenous control according to the manufacturer’s manual (Applied Biosystems 7500 fast-real-time-PCR System or Quant StudioTM 6 Flex Real-Time PCR systems). For evaluation of target gene expression, the ΔCt as well as the ΔΔCt method was used (all Applied Biosystems, Carlsbad, CA, USA).

The following primers were used: mIL-33 (fw 5′CTGCAAGTCAATCAGGCGAC 3′, rv 5′ TGCAGCCAGATGTCTGTGTC 3′) β-Actin (fw 5′ TGTCCACCTTCCAGCAGATGT 3′, rv 5′ AGCTCAGTAACAGTCCGCCTAGA 3′) mIL-1β (fw 5′ CAGGCAGGCAGTATCACTCA 3′, rv 5′ AGGTGCTCATGTCCTCATCC 3′) mIL-6 (fw 5′ TCCATCCAGTTGCCTTCTTG 3′, rv 5′ TTCCACGATTTCCCAGAGAAC 3′) mIL-19 (fw 5′ GCCAACTCTTTCCTCTGCGT 3′, rv 5′ GGTGGCTTCCTGACTGCAGT 3′) mIL-5 (fw 5′ AGCACAGTGGTGAAAGAGACCTT 3′, rv 5′ TCCAATGCATAGCTGGTGATTT 3′) mIL-10 (fw 5′ ACTGCACCCACTTCCCAGT 3′, rv 5′ TTGTCCAGCTGGTCCTTTGT 3′) mIL-4 (fw 5′ AGATGGATGTGCCAAACGTCCTCA 3′, rv 5′ AATATGCGAAGCACCTTGGAAGCC 3′) mIL-13 (fw 5′ TGAGGAGCTGAGCAACATCACACA 3′, rv 5′ TGCGGTTACAGAGGCCATGCAATA 3′) mCCR6 (fw 5′ CTGCAGTTCGAAGTCATC 3′, rv 5′ GTCATCACCACCATAATGTTG 3′) mTNFα (fw 5′ GCTGAGCTCAAACCCTGGTA 3′, rv 5′ CGGACTCCGCAAAGTCTAAG 3′) BD4 (fw 5′ GGCTTCAGTCATGAGGATCCAT 3′, rv 5′ TTTGGGTAAAGGCTGCAAGTG 3′) BD14 (fw 5′ GTGGCCGGTGTGCTGTACT 3′, rv 5′ CGCTATTAGAACATCGACCTATTTGT 3′) mLCN2 (fw 5′ TGGAAGAACCAAGGAGCTGT 3′, rv 5′ GGTGGGGACAGAGAAGATGA 3′).

### ELISA

ELISA was performed with ELISA Kits (eBioscience, San Diego, CA, USA), according to the manufacturer’s instructions. Absorbance was measured at λ450/540 nm with SpectraMax 190 ELISA-Reader and analyzed with Softmax Pro Version 3.0 (Molecular Devices) software. IL-33 ELISA detects mature recombinant IL-33 (rIL-33) and processed forms of full length IL-33 (16).

### Statistical Analysis

Differences between groups were evaluated by unpaired two-tailed *t*-test. We applied the Bonferroni correction for multiple testing. For *in vivo* radiation experiments, differences were evaluated using two 2 × 2 ANOVAs, each incorporating time as a within-subjects characteristic (difference between first measurement at day 2 and the last measurement at day 25) and group assignment as a between-subjects characteristic (control mice vs. K14 IL33 mice). *p* < 0.05 was considered to be significant.

## Results

### Inflammatory Skin Phenotype Induced by Overexpression of Mature IL-33 in K14-CreER™/GFPmmIL33 Mice

**Figure 1:**
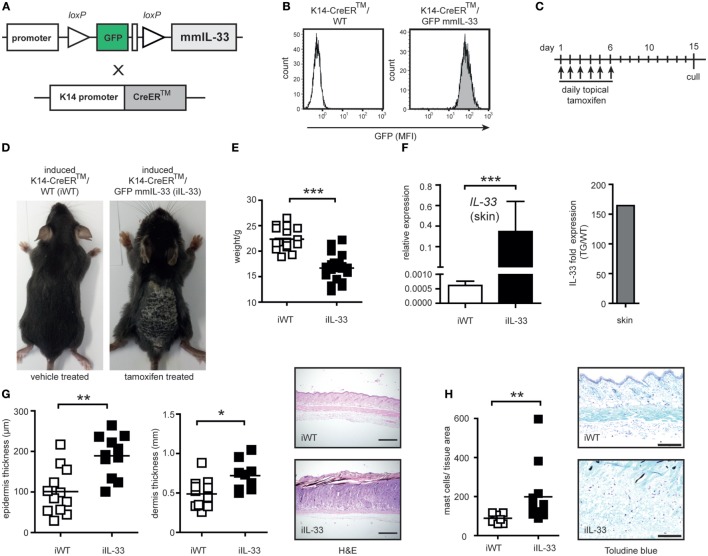
Generation and characterization of the skin-specific K14-CreERTM/GFPmmIL33 mouse overexpressing mature IL-33. **(A)** Schematic illustration of the generation of skin-specific mouse mature (mm) IL-33 expression constructs. Mouse-containing transgene DNA with loxP-flanked GFP and STOP codon which prevents transcription of the mature form of mouse IL-33 cDNA was crossed with tamoxifen (TM) inducible K14-CreERTM mouse. **(B)** The genotyping was performed using flow cytometric analysis of blood for GFP expression. MFI, mean fluorescence intensity. **(C)** Topical administration every day with 20 mg tamoxifen in a total volume of 200 µl (“induced”) results in Cre-mediated recombination with the deletion of the loxP-flanked GFP and STOP codon and strong induction of IL-33 expression under the K14 promoter in keratinocytes. **(D)** Cutaneous manifestations of induced K14-CreERTM/GFPmmIL33 (iIL-33) mice compared to control induced K14-CreERTM/WT (iWT). **(E)** Reduction of weight of iIL-33 mice compared to iWT after the administration of tamoxifen. **(F)** Expression of the mmIL-33 gene in iIL-33 mice relative to iWT mice and fold skin expression of the mmIL-33 gene in iIL-33 mice (Tg/WT; transgene compared to wild-type). Total RNA from skin of mice was used as template for quantitative real-time PCR. On day 15, after the tamoxifen administration, **(G)** the skin was analyzed for epidermal, dermal thickness (μm), and collected for H&E (4× magnification) and **(H)** toluidine blue staining (10× magnification). Toluidine blue-positive mast cells were increased in the lesional skin of iTg compared to iWT after the administration of tamoxifen and results shown of *n* = 9 (WT) and *n* = 12 (Tg). *p < 0.05, **p < 0.01, ***p < 0.0001 (Mann-Whitney).

**Figure 2:**
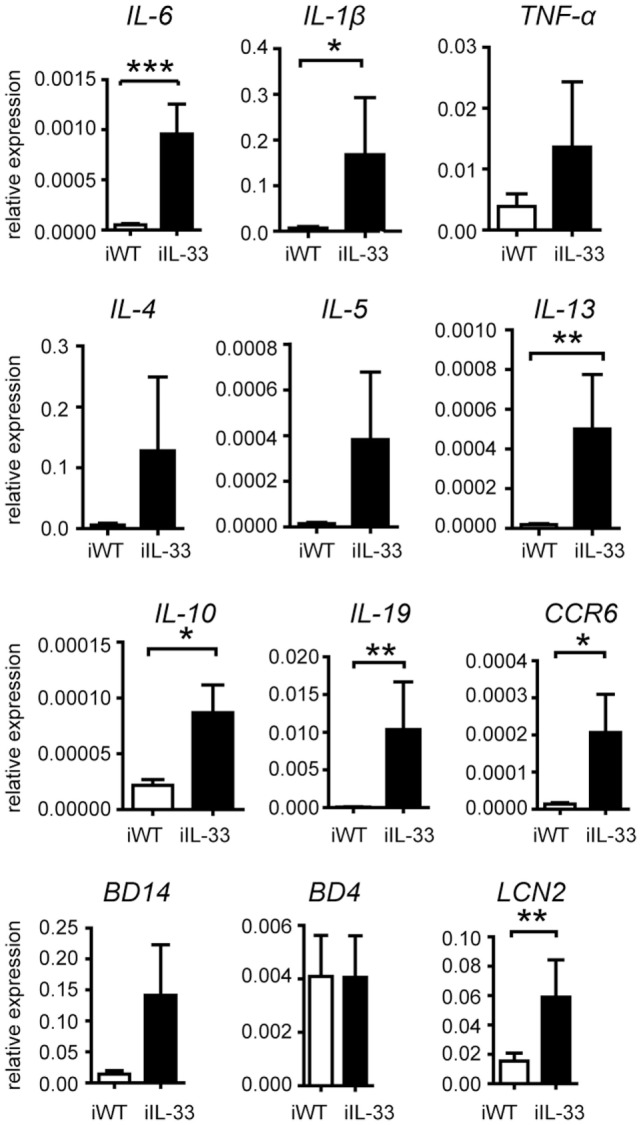
Changes in cytokine profiles of lesional skin of iTg. cDNA isolated from the lesional skin of iTg compared to iWT after the administration of tamoxifen was analyzed for the expression of the respective genes by quantitative real-time PCR. All data are given as mean ± SEM. *p < 0.05, **p < 0.01, and ***p < 0.001 (Mann-Whitney). Three independent experiments.

To test, if mature IL-33 exclusively linked to keratinocytes induce spontaneous inflammation we used an inducible K14-IL-33-CRE™/GFPmmIL33 mouse (iTG). This mouse contains a transgene DNA with loxP-flanked GFP and stop codon, which prevents transcription of the mature form of mouse IL-33 (mmIL-33) cDNA and was crossed with a tamoxifen inducible K14-CreER™ mouse (Figures 1A,B). For the Cre-mediated recombination and K14-specific overexpression of the mmIL-33, tamoxifen (TM) was administrated for 6 days topical on the shaved back skin (Figure 1C). Five days after the last TM administration, adult iTG mice developed thickening and scaling of the skin as well as weight loss (Figures 1D,E). In induced skin, IL-33 expression increased about 160-fold (Figure 1F). Histological analysis of skin biopsies by hematoxylin and eosin staining revealed an increase in epidermis and dermis thickness in iTG compared to WT mouse (Figure 1G). Abundant infiltrates of mast cells were detected by toluidine blue staining (Figure 1H). Furthermore, IL-33 overexpression in skin, lead to high production of the cytokines IL-6, IL-1β, IL-13, IL-10, IL-19, CCR6, and LCN2 (Figure 2).

### Generation and Characterization of Transgenic Mice with Skin-Specific Expression of Full Length IL-33

**Figure 3:**
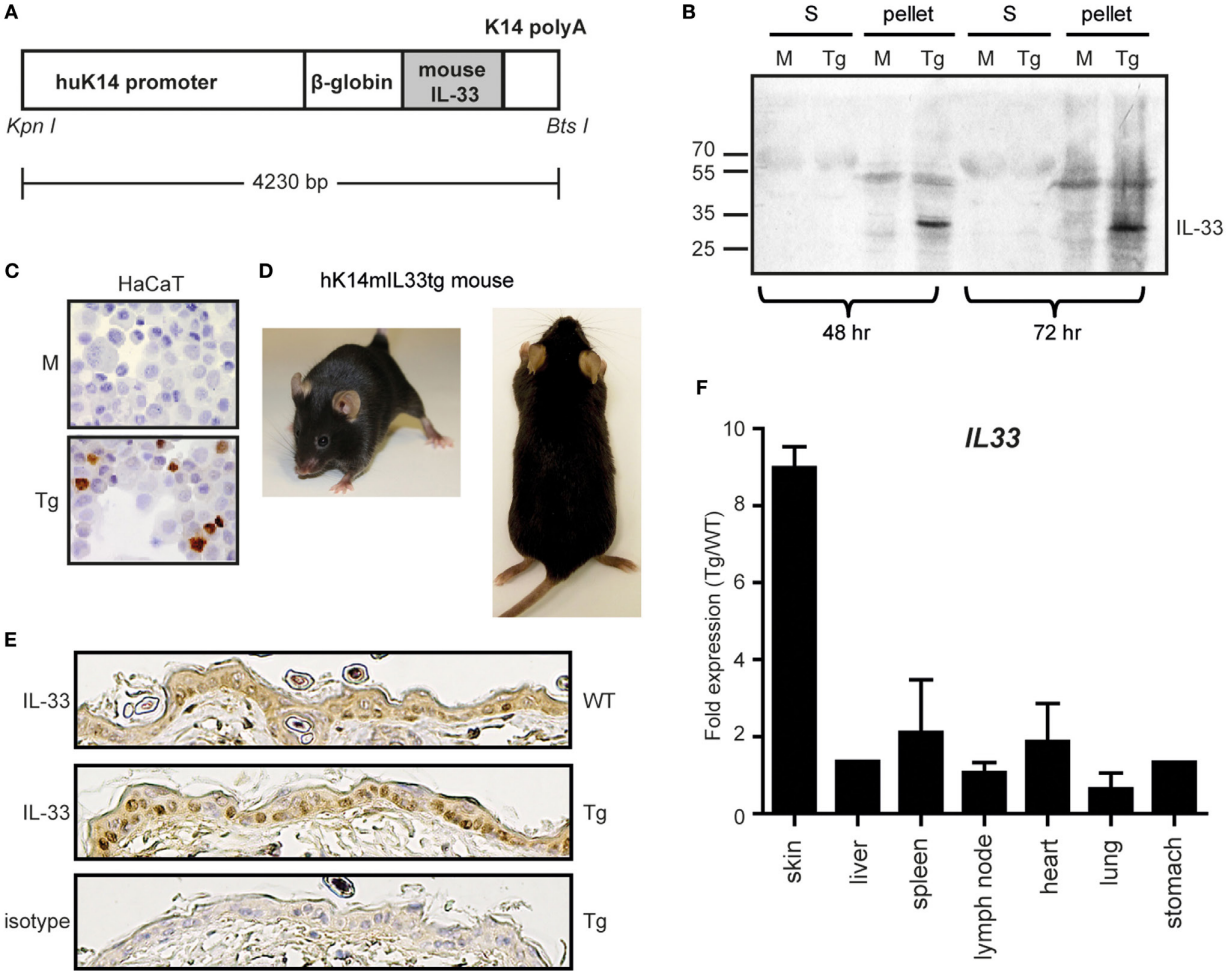
Overexpression of the IL-33 gene in cultured keratinocytes and the skin selective hK14mIL33tg mouse. **(A)** Schematic representation of the transgene DNA. An active full-length form of mouse IL-33 cDNA was placed downstream of the human keratin 14 promoter. The transgene construct also contained a rabbit β-globin intron sequence and a keratin 14 polyadenylation signal for the stable processing of the transcripts. **(B)** Transfection of the human keratinocyte cell line HaCaT with mock (M) or hK14mIL-33 (Tg). After 48 h or 72 h, supernatant (S) and cell pellets were stained for IL-33. **(C)** Immunhistochemistry of hK14mIL-33 or mock transfected HaCaTs. Nuclear staining in the lower panel (DAB, brown dye 40×). **(D)** No cutaneous manifestations of hK14mIL33tg mice up to 6 months. **(E)** Immunhistochemistry of IL-33 in the epidermis of wild-type (WT) and hK14mIL33tg (Tg) back skins. **(F)** The skin-selective expression of the IL-33 gene in hK14mIL33tg mice. Total RNAs from various organs of mice were used as templates for quantitative real-time PCR. Each bar shows the expression of the IL-33 gene in hK14mIL33tg mice relative to WT mice, data represent mean and SEM of *n* = 3 (except liver and stomach; *n* = 1). Other data are representative of three independent experiments.

In order to assess the role of IL-33 in skin inflammation, we developed transgenic mice that expressed the full-length mouse IL-33 under the control of a human K14 promoter *via* pronuclear injection of K14-IL-33 construct (hK14mIL33tg, Figure 3A). To test the functionality of this construct, HaCaT cells were transfected with the plasmid containing hK14mIL33tg or mock control. Lysates and supernatants were tested for IL-33 expression using Western blot analysis (Figure 3B) and IHC (Figure 3C). IL-33 (30 kDa) was strongly expressed in cell lysates after 48 h and 72 h (Figure 3B). IHC showed nuclear expression in transfected HaCaT cells compared to the mock control (Figure 3C). The transgenic mouse line expressing the hK14mIL33tg was generated as described above. Two founder mice were tested positive for the transgene and used as hK14mIL33tg mouse line, all of which had a similar IL-33 expression in the skin. The studies reported in this work were performed in hK14mIL33tg with at least eightfold higher mRNA levels in skin relative to the endogenous IL-33 mRNA level (Figure 3F). To generate littermates, female heterozygous hK14mIL33tg mice were crossed with male WT C57BL/6 mice. The hK14mIL33tg mice grew normally and did not develop phenotypic abnormalities (Figure 3D), treatment with topic tamoxifen did not result in skin changes (data not shown). Skin IHC staining for IL-33 revealed higher expression in transgenic compared to WT mice; however, no skin pathology was observed (Figures 3D,E).

### No Spontaneous or Triggered Cutaneous Inflammation due to Skin-Specific Overexpression of Full Length IL-33

**Figure 4:**
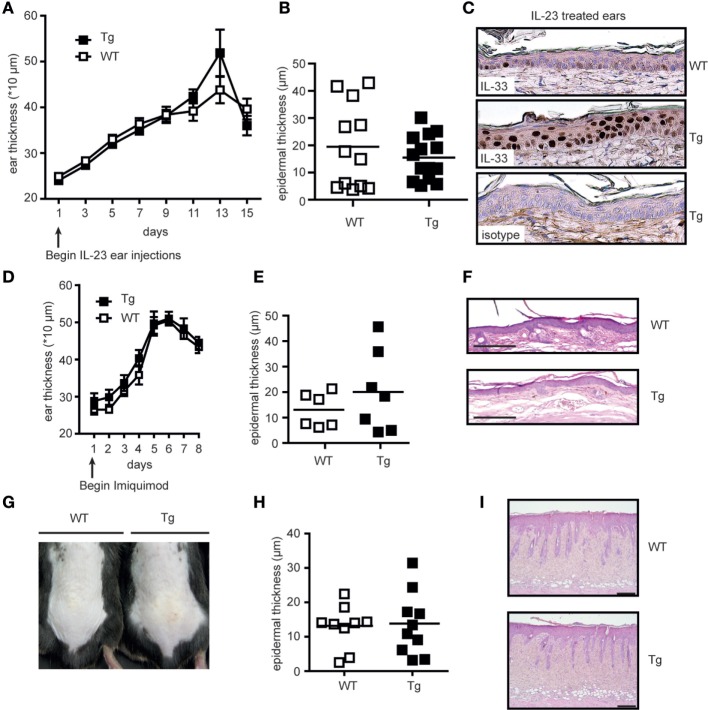
Local skin overexpression of the full length IL-33 does not worsen triggered skin inflammation. Ears from wild-type (WT) and hK14mIL33tg (Tg) were injected intradermally every other day with 500 ng IL-23 in a total volume of 20 µL. **(A)** Ear thickness was measured before each injection. On day 15, ears were **(B)** analyzed for epidermal thickness (μm) and **(C)** collected for immunostaining and results shown are representative of two independent experiments. Overexpression of IL-33 in mice keratinocytes does not worsen the development of IMQ-induced psoriasiform dermatitis. Ears of both WT and Tg mice (*n* > 4 per group) were topically treated with an IMQ-containing cream (Aldara, MEDA Pharma) for 7 consecutive days. **(D)** Ear swelling was measured daily before treatment. On day 7, ears were collected and analyzed for **(E)** epidermal thickness (μm) and **(F)** inflammation by H&E staining (4× magnification). **(G)** Skin barrier disruption in WT and Tg back skins by tape stripping. On day 3 after treatment, back skin of WT and Tg were collected for **(H)** H&E and **(I)** analyzed for epidermal thickness (μm).

The obvious lack of a spontaneous skin phenotype in hK14mIL33tg mice showed that a mere increase in intracellular IL-33 levels was not sufficient to induce local inflammation. This observation in turn indicated that additional signals and events such as an inflammatory trigger or tissue damage are necessary to allow secretion and/or action of full-length IL-33. We therefore aimed to elucidate whether a local inflammatory stimulus was sufficient to release and activate full-length IL-33 in the hK14mIL33tg mouse and performed different skin inflammation models including rIL-23 ear injection, topical IMQ, and barrier disruption using skin tape stripping. The morphology of IL-23-injected skin is similar to lesional skin of human psoriasis (33, 34); therefore, we used an IL-23 ear injection model to estimate the effect of IL-33 overexpression during skin inflammation. rIL-23 was intradermally injected in the ear of hK14mIL33tg and WT mice every other day. Ear thickness and histological analysis such as epidermal thickness were used as readout (Figures 4A–C). Measurement of ear and epidermal thickness did not reveal any difference between hK14mIL33tg and WT mice. IHC and HE staining of ear sections showed that transgenic mice express more nuclear IL-33 compared to WT mice (Figure 4C), but showed no difference in epidermal hyperplasia. To assess whether overexpression of IL-33 in mice keratinocytes worsens the development of IMQ-induced psoriasiform dermatitis, we applied IMQ cream on the right ear of hK14mIL33tg and WT mice for 7 consecutive days. Daily measurements of ear thickness but also histological analysis did also not show any difference in inflammation comparing hK14mIL33tg to WT mice (Figures 4D–F). Furthermore, no difference was observed using a barrier disruption method (Figures 4G–I). In comparison to the iTG mice, we also treated hK14mIL33tg mice with tamoxifen; similar to iWT mice (Figure 1D), no effect was observed (data not shown). Thus, despite different immunological provocations no phenotype of full length (fl) IL-33 overexpression could be observed, which showed that skin inflammation *per se* does not promote action of IL-33.

### Induced Dermatitis in hK14mIL33tg Mice after Fractionated Radiation

**Figure 5:**
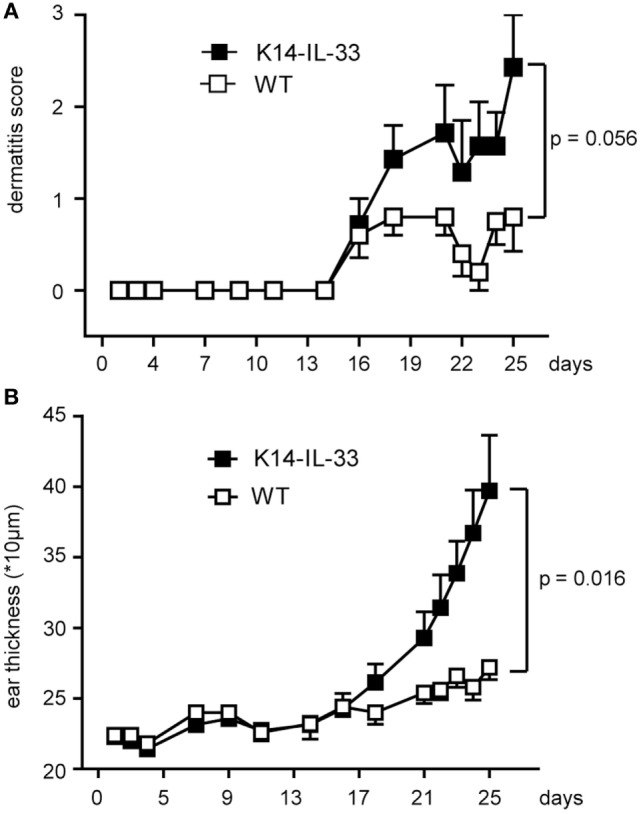
Full length IL-33 exacerbates irradiation-induced dermatitis. hK14mIL33tg (K14-IL-33) mice were locally irradiated with four single fractions of 12 Gy (cumulative dose of 48 Gy, 6 MV, right ear). This procedure was performed at days 1, 3, 5, and 7 and dermatitis **(A)** (K14-IL-33 *n* = 15, WT *n* = 10) and ear thickness **(B)** (K14-IL-33 *n* = 7, WT *n* = 5) measured as indicated. Two 2 × 2 ANOVAs, each incorporating time as a within-subjects characteristic (difference between first measurement at day 2 and the last measurement at day 25) and group assignment as a between-subjects characteristic (control mice vs. K14 IL33 mice) suggested a significant interaction of time and group assignment for ear thickness (*p* = 0.016) and a near significant result in view of the clinical score (*p* = 0.056). These results were retrieved besides the significant single effect of time in both models (*p* ≤ 0.002).

**Figure 6:**
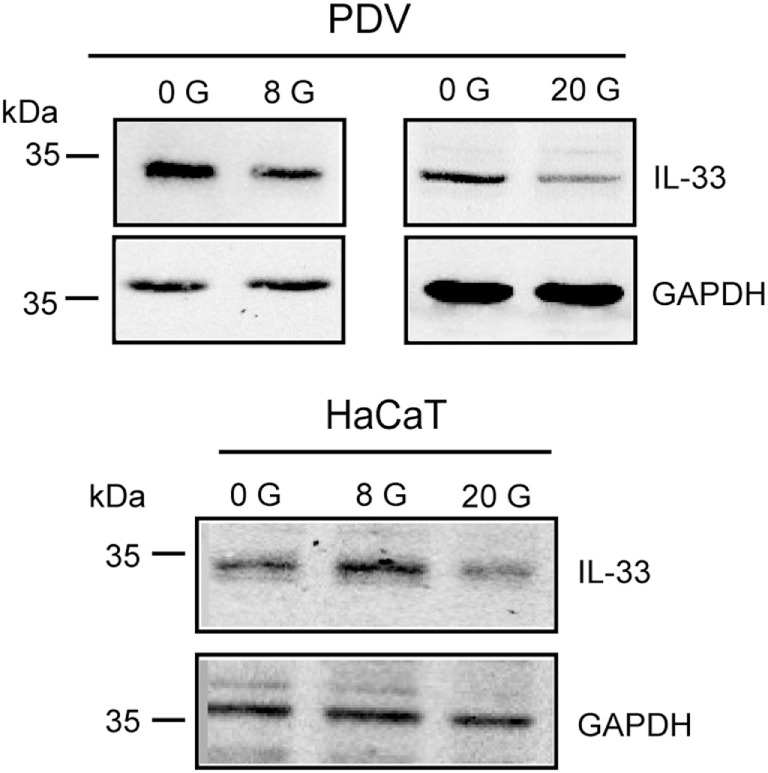
Expression of IL-33 by radiation in keratinocytes. Intracellular IL-33 expression in lysates of murine (PDV) and human keratinocyte cell line (HaCaT) 24 h after irradiation at 0 (unirradiated), 8 Gy as well as 20 Gy was analyzed by Western blot. GAPDH served as loading control. Data are representative of at least three independent experiments.

In the here reported mouse models, only secreted mmIL-33 led to a clinically relevant phenotype. In serum analysis, IL-33 was also only clearly expressed in the induced K14-mmIL-33-CRE-TM (Figure S1 in Supplementary Material). Fl IL-33 has been demonstrated to be released from damaged cells (15). As radiation induces a strong dermatitis due to apoptosis and necrosis, we next determined the effect of fractionated radiation (single fraction 12 Gy, 4 fractions within 7 days) in hK14mIL33tg and WT mice (Figures 5A,B). In comparison to WT mice, an increase in ear thickness in hK14mIL33tg was observed 25 days after irradiation (Figure 5B). Macroscopic examination of irradiated ears indicated that transgenic mice have more severe skin symptoms compared to WT mice (Figure 5A). To confirm that IL-33 is affected in irradiated keratinocytes *in vitro*, we used the murine keratinocyte cell line PDV and the HaCaT transfected with the K14-flIL-33 vector and irradiated the cells. Radiation with 20 Gy induced an intracellular decrease of IL-33 in both cell lines (Figure 6). In conclusion, IL-33 contributed to a skin radiation-induced phenotype.

## Discussion

Various members of the IL-1 cytokine family have been implicated in inflammation in a variety of tissues and diseases (11). Here, we demonstrated that IL-33 has the potential to induce skin inflammation with high potency if present in its mature form. Full length expression of IL-33, in turn, was not able to induce spontaneous inflammation or boost an ongoing inflammatory reaction, but rather sensitized the tissue for radiation damage. With induction of maturated IL-33 in the skin of K14-mmIL-33 mice, cytokines responsible for skin inflammation were upregulated. These included pro-inflammatory cytokines such as IL-6 and TNF but also Th2 cytokines. As initial responder tissue resident innate lymphoid cells 2 and mast cells, both expressing ST2, may be crucial for inflammation initiation (35–40). Also LCN2, which is induced in DCs by IL-33 and involved in the Th2 polarization process has been upregulated (41). But also hematopoietic cells such as regulatory T cell (Tregs), Th2 cells, eosinophils, basophils that also constitutively express high levels of ST2 could account for a systemic inflammation. The systemic phenotype was supported by detection of serum IL-33 and overall inflammation-mediated disease with pronounced weight loss of affected mice.

As full length, but not mature IL-33 is predominant in healthy skin, we additionally studied the phenotype of K14-mflIL-33 Tg mice. The resulting data provided *in vivo* evidence that overexpression of keratinocyte-derived fl IL-33 is by itself not sufficient for cutaneous inflammation. The epidermis and dermis of these mice were unremarkable even after stimulation with different skin inflammation models such as IL-23 injection, mechanical tissue disruption, and IMQ treatment. Interestingly, a similar generated mouse by Imai et al. was reported to develop spontaneous dermatitis-like inflammation (3). Transgene integration by Imai et al. occurred in 12 mice with increased IL-33 expression in 6 mice. Only two of these developed skin disorders. In comparison, our generation yielded only one high expressing mouse line with no overt phenotype (Figure 3). Fold expression differed from 8-fold (mouse line presented here) to ~24-fold (mouse by Imai et al.). Phenotype differences might be due to different threshold levels of cytokine expression, insertion of the DNA, or disruption of secretion properties. Since in our described model, no IL-33-dependent exacerbation of skin inflammation was observed, we hypothesized that an additional signal is necessary for externalization and action of fl IL-33. A peripheral blood and skin work up during steady state was not performed due to the missing phenotype. However, when massively overexpressing IL-33 (mature form), we demonstrate that mmIL-33 not only induces the Th2 cytokine axis with IL-5 and IL-13 but also Th1/17 cytokines such as IL-6, IL-1, and CCR6 (Figure 2). This could reflect the different properties of IL-33 in regard to reported capacity to influence allergy associated models in comparison to Th1/Th17 dependent arthritis models (42). In the contrary to the high expression, this could be also an effect due to the mature vs. full length form.

Furthermore, comparing the here presented >150-fold upregulation in the K14-mmIL-33-CRE-TM mouse with the eightfold hK14mIL33tg mouse, it is not clear if the skin effect arises from the mature form in contrast to the high expression. Tamoxifen induced mmIL-33 was detected in the serum of mice (Figure S1 in Supplementary Material), whereas IL-33 from fl IL-33 overexpressing mice was not detectable in the serum. Also, the fl IL-33 expressing mouse from Imai et al. (~24-fold) did not show systemic IL-33 in the serum. Thus, it is unclear if the high concentration or the structural form of IL-33 leads to the phenotype in the secreted K14-mmIL-33-CRE-TM. Further work is needed, comparing both forms with similar expression patterns.

A limitation of this work is that we could not differentiate between apoptotic and/or necrotic influences of different challenges in the K14-mflIL-33 mouse. Fl IL-33 is released by necrosis and proposed to function as an “alarmin” (12). Ionizing radiation induces cellular DNA damage leading to a release of “danger signals” and pro-inflammatory cytokines including IL-33 in various tissues such as mouse bone marrow, intestinal cells, spleen, and thymus (43, 44). Also, IL-33 was induced after single dose skin irradiation (45). Radiation of HUVECs (an endothelial cell line) led to IL-33 release in the supernatant (45). Here, using two keratinocyte cell lines transfected with fl IL-33, we observed a decrease of cellular IL-33 after radiation with 20 Gy. PDV cells showed also reduced cellular IL-33 expression post 8 Gy (Figure 6). Furthermore, in our inflammation models, we do not know if different stimuli such as IL-23 trigger endogenous IL-33 expression.

Accordingly, local repeated irradiation of skin in K14-mflIL-33 transgenic mice led to increased thickness and dermatitis. In human skin fibroblasts, radiation-induced IL-33 expression in cell lysates 1 and 4-h postradiation, whereas no IL-33 in supernatant could be measured (46). Interestingly, also bystander cells increased IL-33 protein expression. In comparison, our data suggest that intracellular IL-33 expression decreases after 24 h. Although *in vitro* effects at early time points have been demonstrated by Ivanov et al., *in vivo* irradiation in the K14-mflIL-33 mice only showed clinically long-term changes after 3 weeks (Figure 5). In this notion, cutaneous injection of IL-33 into the skin induced fibrosis and inflammation after 1 week (7, 47). Thus, although a fast local response with IL-33 release and other danger signals occurs, these effects most likely influence later endogenous accumulation of extracellular matrix components. The limitation of our study is the lack of fibrosis work up in the used radiation model.

Interleukin-33-dependent radiation response could potentially be compared with an early tissue insult in tendinopathy. In this disease, a mechanical damage induces release of IL-33 by tenocytes (fibroblasts of tendons) (5). IL-33 drives rapid repair, however, with different regulation of collagen and subsequent decrease in biomechanical quality/strength of the tendon. In cutaneous wound healing, IL-33/ST2 supports cell recruitment as early as 24 h after wounding (48). Transferring these observations IL-33 might lead to early repair/healing effects in irradiated tissue. This is accompanied with recruitment of inflammatory cells, polarized immune responses contributing to skin disease and following fibrotic changes.

Since IL-33 levels vary depending on the tissue and are differently regulated between health and disease more studies are needed to elucidate its role in skin inflammation.

## Ethics Statement

This study was carried out in accordance with the recommendations of federal guidelines (GV-SOLAS). The protocol was approved by the “Regierung von Unter-/Mittelfranken.”

## Author Contributions

OK: performed most of the practical work together with BF, LS, VS, SA, and MH and wrote the manuscript together with SF and AH. BF: contributed to the design of the irradiation procedures, performed the planning of the mouse irradiation protocol and conducted the *in vivo* and *in vitro* irradiation procedures, and contributed to the evaluation of the data and writing of the manuscript. LS: performed the practical work together with OK and BF. VS, AS, MH, and LS: performed the practical work together with OK and BF. SA: performed the practical work together with OK. DG: contributed to the design of the work and construction of the K14-flIL-33 mouse line. UG, GK, and GS: contributed to the evaluation of the data and the writing of the manuscript. IM: contributed to the design of the work. SW: contributed to the design of the work and provided the tamoxifen inducible mmIL-33-GFP-CRE mouse. SF: drafted and designed the study together with AH, advised experiments of OK, LS, VS, SA, and MH; contributed to the evaluation of the data and writing of the manuscript. AH: drafted and designed the study, drafted the manuscript, and wrote it together with SF and OK.

## Conflict of Interest Statement

The authors declare that the research was conducted in the absence of any commercial or financial relationships that could be construed as a potential conflict of interest.
