# Supplementary figures and images for: Full Length Interleukin 33 Aggravates Radiation-Induced Skin Reaction
Source: Front Immunol. 2017 Jun 28;8:722. doi: 10.3389/fimmu.2017.00722 (PMC5487387; doi:10.3389/fimmu.2017.00722)

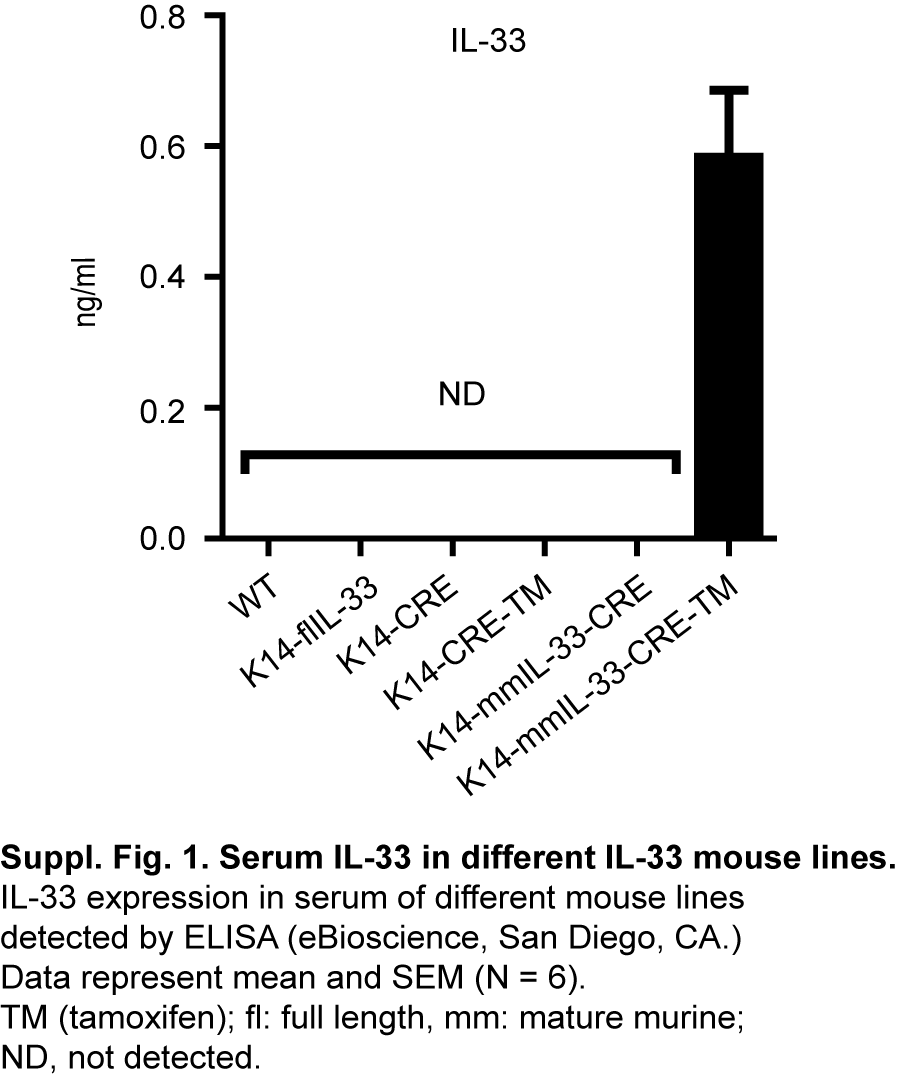

Supplement: Supplementary file 1 [file Image_1.TIF]
